# Comprehensive analysis of the expression and prognosis for TNFAIPs in head and neck cancer

**DOI:** 10.1038/s41598-021-95160-x

**Published:** 2021-08-03

**Authors:** Gaochen Lan, Xiaoling Yu, Xin Sun, Wan Li, Yanna Zhao, Jinjian Lan, Xiaolong Wu, Ruilan Gao

**Affiliations:** 1 Institute of Hematology Research, The First Affiliated Hospital of Zhejiang Chinese Medical University, 54 Youdian Road, Hangzhou, 310006 China; 2 Department of Oncology, Zhejiang Provincial People’s Hospital, Hangzhou, 310014 China

**Keywords:** Head and neck cancer, Prognostic markers

## Abstract

Head and neck cancer (HNC) tumorigenesis involves a combination of multiple genetic alteration processes. Tumour necrosis factor-alpha-induced proteins (TNFAIPs) are involved in tumour development and progression, but few studies have been conducted on these factors in HNC. We aimed to analyse TNFAIPs and assess their potential as prognostic biomarkers and therapeutic targets using the Oncomine, UALCAN, Human Protein Atlas, LinkedOmics, cBioPortal, GeneMANIA, Enrichr, and Tumor IMmune Estimation Resource databases. We found that the transcript levels of TNFAIP1, TNFAIP3, EFNA1, TNFAIP6 and TNFAIP8 were increased, while those of TNFAIP8L3 and STEAP4 were reduced in HNC tissues versus normal tissues. The EFNA1, TNFAIP8 and TNFAIP8L3 expression levels were significantly correlated with the pathological stage. In HNC patients, high PTX3 and TNFAIP6 transcript levels were significantly associated with shorter overall survival (OS). Moreover, genetic alterations in TNFAIP1, TNFAIP6, and STEAP4 resulted in poorer disease-free survival, progression-free survival, and OS, respectively. TNFAIPs may mediate HNC tumorigenesis by regulating PI3K-Akt, Ras and other signalling pathways. TNFAIPs are also closely correlated with the infiltration of immune cells, including B cells, CD8^+^ T cells, CD4^+^ T cells, etc. The data above indicate that TNFAIPs may be potential biomarkers and therapeutic targets for HNC.

## Introduction

Head and neck cancer (HNC) is the seventh most common malignancy worldwide, with more than 890,000 new cases and 450,000 deaths per year^1,2^. HNC includes tumours arising in the lip, mouth, paranasal sinus, oropharynx, nasopharynx and hypopharynx. Squamous cell carcinoma is the histologic type in more than 90% of HNC cases. Surgery, chemotherapy, and radiotherapy are the main treatments for HNC, and great progress in these fields has been made in recent years, which has improved the long-term survival rates of early-stage patients to approximately 70–90%^1^. However, 5-year survival rate remains less than 40% in advanced or metastatic patients^3^. In addition, routine therapies may affect organ function and damage structures related to speaking and swallowing, causing a severe decline in the quality of life of HNC patients. Therefore, new therapies are needed to compensate for the shortcomings of conventional treatments. At present, the prognosis of HNC mainly depends on the tumour-node-metastasis (TNM) stage. However, the TNM stage is based on anatomical information and does not reflect the biological heterogeneity of HNC. Hence, it is urgent to find new biomarkers that can act as prognostic indicators to guide precise individualized treatment. In recent years, researches on targeted therapy have been performed, and PD-1 immune checkpoint inhibitors have been developed^4,5^. The anti-PD-1 antibodies pembrolizumab and nivolumab showed durable responses and survival improvements in patients with recurrent or metastatic HNC, leading to approval of these two agents by the FDA in 2016^1,6^. However, immunotherapy is only suitable for some patients and thus cannot meet clinical needs. Therefore, more therapeutic targets and prognostic biomarkers should be identified.

Tumour necrosis factor-alpha-induced proteins (TNFAIPs) were initially identified as tumour necrosis factor-alpha- and lipopolysaccharide-induced genes in endothelial cells^7–9^. The TNFAIP family members include TNFAIP1, TNFAIP2, TNFAIP3, EFNA1, PTX3, TNFAIP6, TNFAIP8, TNFAIP8L1, TNFAIP8L2, TNFAIP8L3, and STEAP4, which are involved in immune reactions, inflammatory responses, signal transduction, apoptosis, differentiation, material transport and other biological functions. They play important roles in multiple diseases, especially malignant tumours^8,10,11^. Several TNFAIPs are deeply involved in the immune and inflammatory processes of cancer. For example, TNFAIP1 induces cervical cancer cell apoptosis by inhibiting the NF-κB signalling pathway^12^. Inflammation induces copper uptake via the STEAP4/IL-17/XIAP axis, which promotes the tumorigenesis and progression of colon cancer^13^. PTX3 regulates complement system activation by interacting with complement regulator H, resulting in C3a- and C5a-mediated recruitment of macrophages and promotion of IL-1β and IL-17 secretion, which leads to the inflammatory activation of malignant disease^14^. Moreover, PTX3 is also a key target of the NF-κB signalling pathway and regulates inflammatory processes in breast cancer^15^. Previous studies found that TNFAIP8 was overexpressed in CD4^+^ and CD8^+^ T cells in patients with lung cancer^16^, suggesting that TNFAIP8 might be involved in the regulation of tumour immune status. It has been reported that differentially expressed TNFAIPs have prognostic value in multiple malignant diseases^17–19^. Zhang et al.^17^ reported that overexpression of TNFAIP1 was associated with a poor prognosis in breast cancer. TNFAIP2 highly expressed in classic Hodgkin's lymphoma and mediastinal diffuse large B-cell lymphoma and is associated with the progression of these malignant diseases^20^. In addition, EFNA1 expression is upregulated in gastric cancer, oesophageal squamous cell carcinoma, hepatocellular carcinoma, cervical cancer and ovarian cancer^21–25^ and is positively correlated with a poor prognosis.

The expression profiles of several TNFAIPs in HNC have been reported in previous studies^26–28^. However, the underlying mechanism by which TNFAIPs are activated or inhibited and the distinct functions of TNFAIPs in HNC have yet to be fully elucidated. Therefore, it is of great significance to clarify the value of TNFAIPs as prognostic biomarkers and therapeutic targets for HNC. In this study, we used several large databases to analyse the expression of TNFAIPs and assess their potential as prognostic biomarkers to provide a scientific basis for personalized medicine for HNC.

## Results

### Differentially expressed TNFAIPs in HNC patients

**Figure 1:**
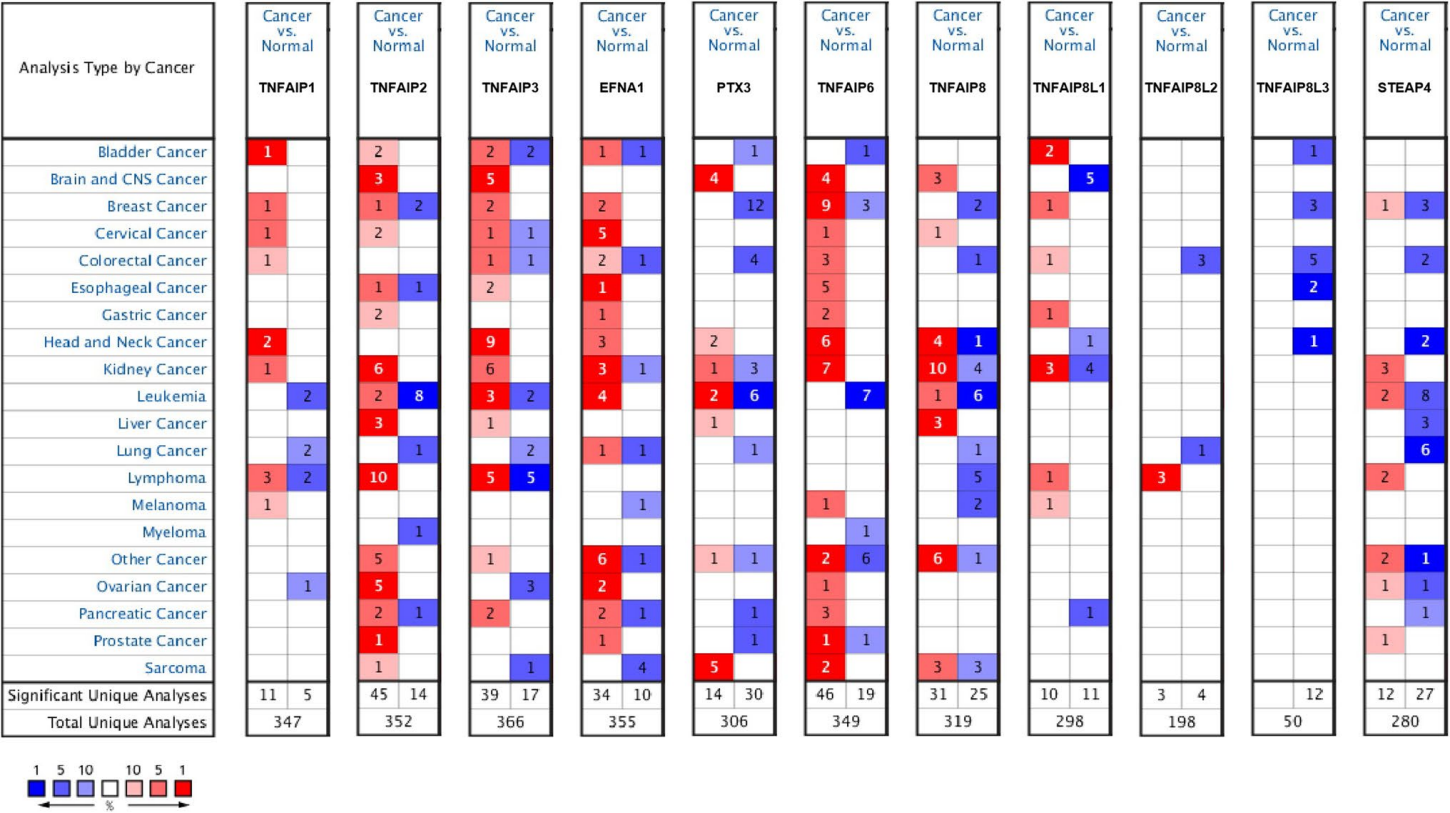
mRNA levels of TNFAIPs in HNC. The figure shows the numbers of datasets with statistically significant upregulation (red) or downregulation (blue) of TNFAIPs at the mRNA level (Oncomine).

**Figure 2:**
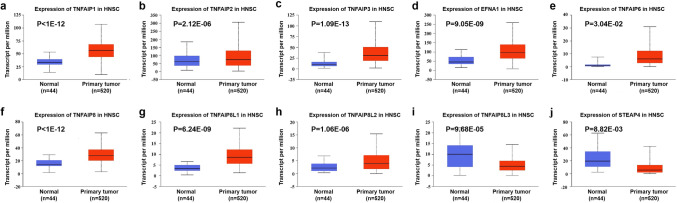
Comparison of the transcript level of TNFAIPs between HNC tissues and normal tissues. The transcript levels of (**a**) TNFAIP1, (**b**) TNFAIP2, (**c**) TNFAIP3, (**d**) EFNA1, (**e**) TNFAIP6, (**f**) TNFAIP8, (**g**) TNFAIP8L1, and (**h**) TNFAIP8L2 were significantly upregulated, while (**i**) TNFAIP8L3 and (**j**) STEAP4 were significantly downregulated in HNC tissues versus normal tissues (UALCAN).

We explored the transcript levels of eleven TNFAIPs in HNC and normal tissues using Oncomine. The results are presented in Fig. 1 and Table 1. According to the results above, the transcript levels of TNFAIP1, TNFAIP3, EFNA1, PTX3, TNFAIP6, and TNFAIP8 were significantly upregulated, while the transcript levels of TNFAIP8L1, TNFAIP8L3, and STEAP4 were significantly downregulated in HNC tissues compared with normal tissues (all *P* < 0.05). Dataset-based analysis of cancer versus normal samples showed that TNFAIP1 expression was increased with fold changes of 2.230 and 2.190 in 31 cases of tongue squamous cell carcinoma tissues and 15 cases of tongue carcinoma^26,29^. The expression of TNFAIP3 was upregulated by a fold change of 2.051 to 5.457 in tissues from 247 patients with HNC in nine datasets^26–31^. EFNA1 expression was increased 2.329-fold to 5.986-fold in HNC tissues from 68 patients^29,30,32^. PTX3 was also overexpressed with fold changes of 4.481 and 3.883 in tissues from 34 and 41 patients with HNC, respectively^27,30^. Analysis of six datasets revealed that TNFAIP6 expression was increased by a fold change of 2.047 to 4.998 in 193 patients^27,28,30,33–35^. Moreover, TNFAIP8 expression increased by 2.427-fold to 3.899-fold in tissues from 76 patients with HNC^26,29,31^. In contrast, the expression levels of TNFAIP8L1, TNFAIP8L3, and STEAP4 were downregulated, with fold changes of 2.735, 2.233, and 2.587, respectively, in 94 HNC samples^26,28,34^. However, the transcript levels of TNFAIP2 and TNFAIP8L2 were not significantly different between cancer samples and normal samples. We further evaluated the expression levels of TNFAIPs in 520 HNC and 44 normal samples by the UALCAN database. As expected, the transcript levels of TNFAIP1, TNFAIP2, TNFAIP3, EFNA1, TNFAIP6, TNFAIP8, TNFAIP8L1 and TNFAIP8L2 were significantly elevated in HNC samples versus normal samples, while the transcript levels of TNFAIP8L3 and STEAP4 were significantly reduced (Fig. 2, all *P* < 0.05).

**Table 1:** The mRNA levels of TNFAIPs in different types of HNC tissues and normal tissues (Oncomine).

TNFAIPs	Types	Fold change	*P* value	*T* test	References
TNFAIP1	Tongue squamous cell cancer	2.230	4.25E−12	8.643	^29^
	Tongue cancer	2.190	2.63E−6	5.511	^26^
TNFAIP3	Tongue squamous cell cancer	3.418	7.44E−12	8.479	^29^
	Head and neck squamous cell cancer	4.217	3.11E−14	11.572	^27^
	Oropharyngeal cancer	3.778	8.17E−6	6.531	^26^
	Tongue cancer	3.160	6.17E−6	5.740	^26^
	Tonsillar cancer	3.132	0.006	3.553	^26^
	Tongue squamous cell cancer	2.051	2.04E−10	7.550	^31^
	Head and neck squamous cell cancer	5.457	2.30E−4	7.760	^30^
	Oral cavity squamous cell cancer	2.865	1.43E−13	10.318	^28^
	Tongue squamous cell cancer	2.385	4.49E−5	4.427	^35^
EFNA1	Tongue squamous cell cancer	5.986	8.61E−9	6.893	^29^
	Head and neck squamous cell cancer	2.619	0.002	4.354	^30^
	Tongue squamous cell cancer	2.329	0.033	3.342	^32^
PTX3	Head and neck squamous cell cancer	4.481	0.005	3.684	^30^
	Head and neck squamous cell cancer	3.883	4.40E−6	5.215	^27^
TNFAIP6	Nasopharyngeal cancer	4.998	2.94E−13	10.617	^34^
	Tongue squamous cell cancer	2.904	6.54E−6	5.146	^35^
	Thyroid gland undifferentiated cancer	2.047	0.003	5.139	^33^
	Head and neck squamous cell cancer	2.816	1.19E−8	6.623	^27^
	Head and neck squamous cell cancer	4.623	0.006	4.934	^30^
	Oral cavity squamous cell cancer	2.367	2.57E−9	6.677	^28^
TNFAIP8	Floor of the mouth cancer	3.899	1.05E−7	7.067	^26^
	Oropharyngeal cancer	2.793	2.75E−4	4.460	^26^
	Tongue squamous cell cancer	2.427	4.17E−9	6.924	^31^
	Tongue squamous cell cancer	2.480	2.46E−8	6.566	^29^
TNFAIP8L1	Nasopharyngeal cancer	− 2.735	0.002	− 3.847	^34^
TNFAIP8L3	Oral cavity squamous cell cancer	− 2.233	1.05E−19	− 13.298	^28^
STEAP4	Oropharyngeal cancer	− 2.587	2.00E−6	− 6.067	^26^
	Nasopharyngeal cancer	− 2.553	8.49E−4	− 4.207	^34^

**Figure 3:**
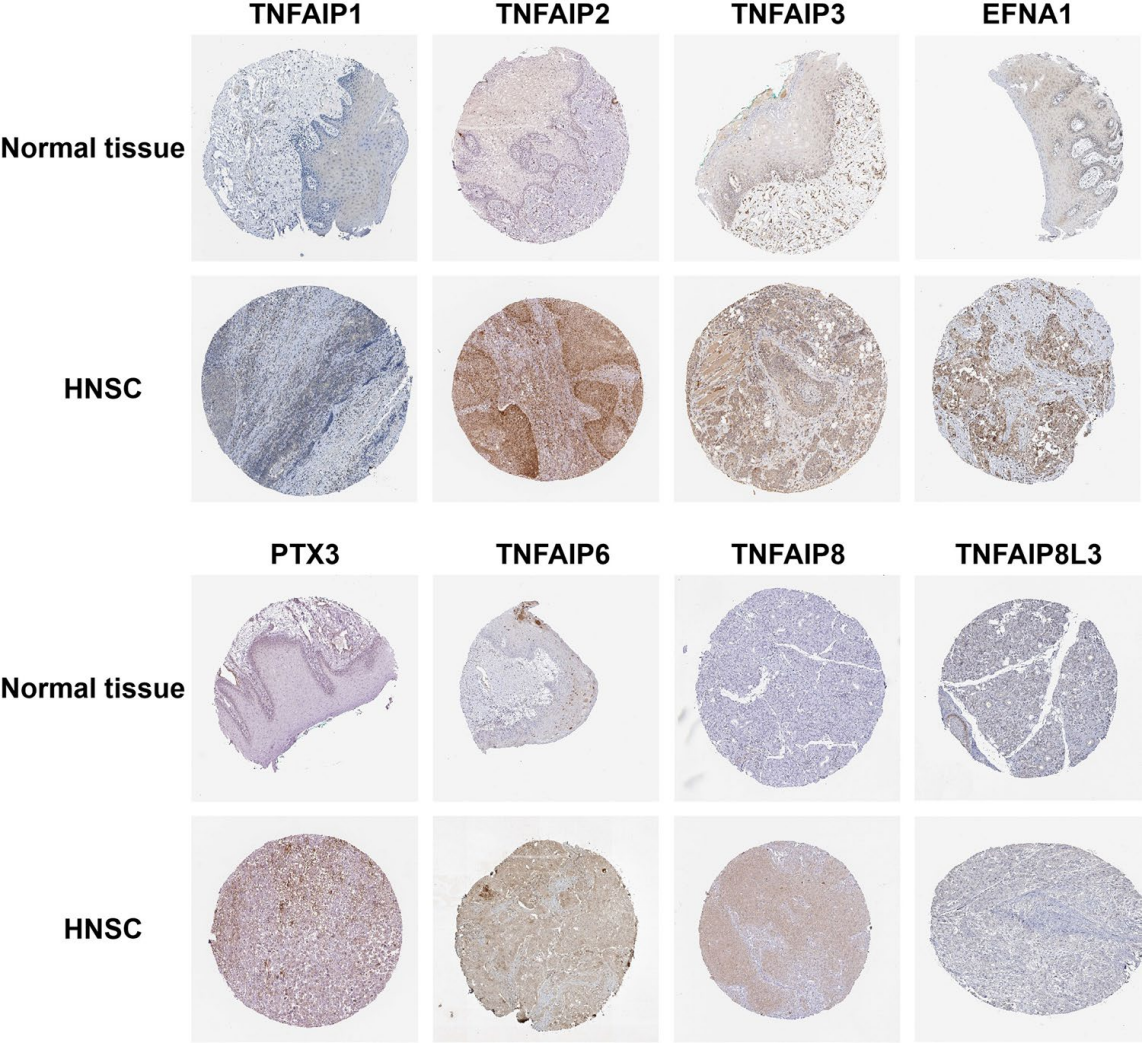
Representative immunohistochemistry images of TNFAIPs in HNSC and normal tissues. The protein levels of TNFAIP2, TNFAIP3, EFNA1, PTX3, TNFAIP6, and TNFAIP8 in HNSC tissues were higher than those in normal tissues (Human Protein Atlas)^52^.

On the other hand, the Human Protein Atlas database was used to evaluate the protein expression levels of TNFAIPs in head and neck squamous cancer (HNSC) and normal tissues by immunohistochemistry, and the results are presented in Fig. 3. Based on the immunohistochemical staining images, the protein expression levels of TNFAIP2, TNFAIP3, EFNA1, PTX3, TNFAIP6 and TNFAIP8 in HNSC samples were higher than those in normal samples, which was consistent with the transcriptomics results.

### The relationship between the mRNA levels of TNFAIPs and clinicopathological parameters in HNC patients

In the LinkedOmics portal, the nonparametric test was performed to compare the mRNA expression of TNFAIPs between patients grouped according to clinicopathological parameters such as T stage (448 patients), N stage (411 patients), M stage (182 patients), pathologic stage (439 patients), and radiotherapy status (451 patients) (Supplementary Table S1). The transcript levels of EFNA1 (*P* = 4.09E−04) were significantly elevated, while the levels of TNFAIP8 (*P* = 3.42E−02), TNFAIP8L2 (*P* = 1.68E−03), and TNFAIP8L3 (*P* = 9.16E−03) was significantly reduced in T3–T4-stage samples compared with T1–T2-stage samples. The expression of TNFAIP8L2 (*P* = 4.39E−02) was increased, while that of TNFAIP1 (*P* = 7.03E−04), TNFAIP8L3 (*P* = 3.12E−02), and STEAP4 (*P* = 3.93E−03) was decreased in node-positive patients compared to node-negative patients. In addition, the expression levels of EFNA1 (*P* = 2.68E−02), TNFAIP8 (*P* = 2.77E−02), and TNFAIP8L3 (*P* = 6.48E−03) showed a significant association with tumour stage. As the tumour stage progressed, the expression of EFNA1 increased, while that of TNFAIP8 and TNFAIP8L3 decreased. Moreover, patients receiving radiotherapy exhibited high expression of TNFAIP2 (*P* = 2.02E−02) and EFNA1 (*P* = 3.60E−03) and low expression of TNFAIP1 (*P* = 2.65E−02), TNFAIP8L3 (*P* = 2.57E−02) and STEAP4 (*P* = 6.05E−03). The results suggest that the expression levels of these TNFAIPs are closely related to the occurrence and development of HNC.

### The impact of TNFAIP expression on the prognosis of HNC patients

**Figure 4:**
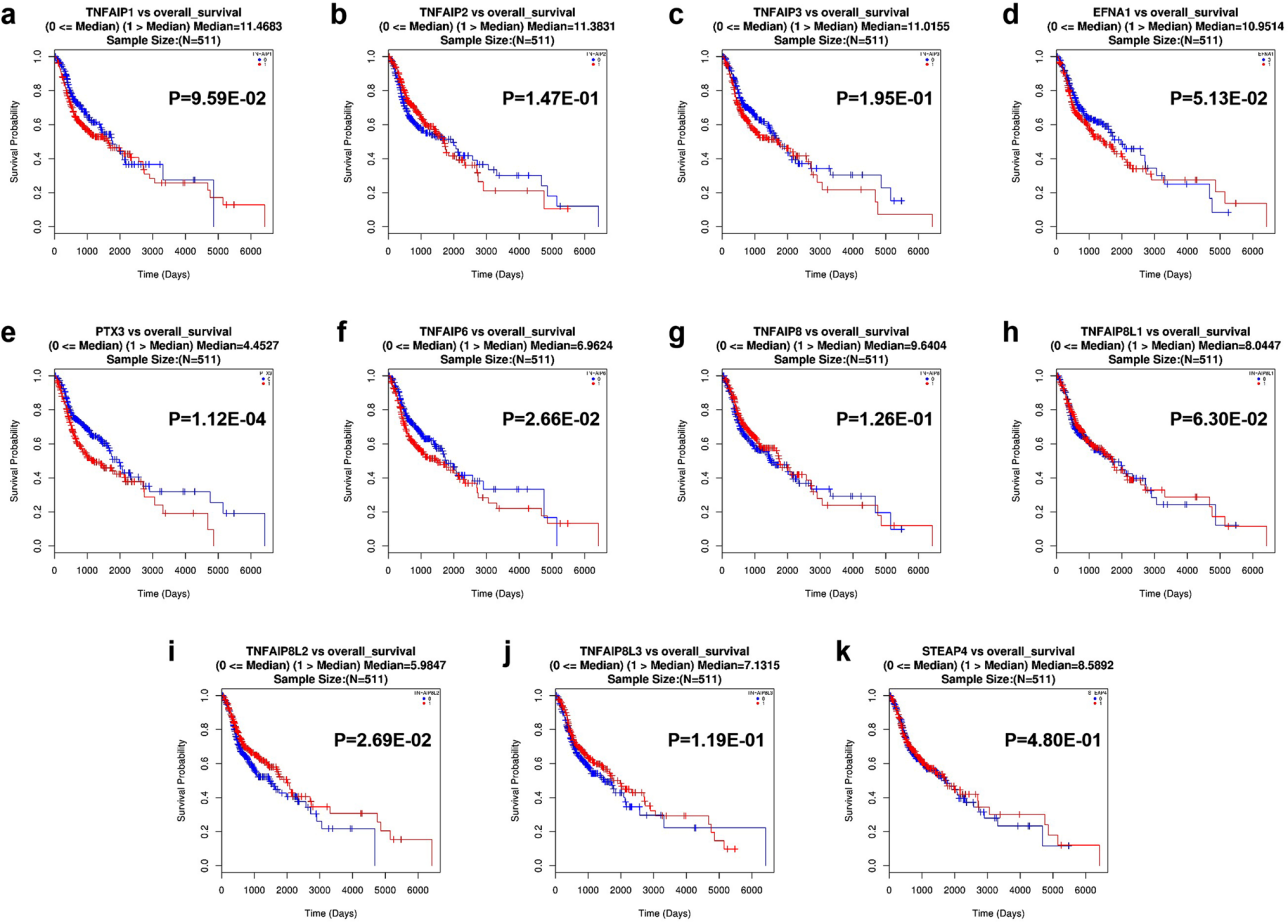
The prognostic value of TNFAIPs in HNC patients. The OS curves of HNC patients according to (**a**) TNFAIP1, (**b**) TNFAIP2, (**c**) TNFAIP3, (**d**) EFNA1, (**e**) PTX3, (**f**) TNFAIP6, (**g**) TNFAIP8, (**h**) TNFAIP8L1, (**i**) TNFAIP8L2, (**j**) TNFAIP8L3, and (**k**) STEAP4 expression (LinkedOmics).

The LinkedOmics portal was utilized to assess the correlation between the mRNA expression of TNFAIPs and the overall survival (OS) of 511 patients with HNC, and the OS curves are shown in Fig. 4. High transcript levels of PTX3 (*P* = 1.12E−4, Fig. 4e) and TNFAIP6 (*P* = 2.66E−2, Fig. 4f) were significantly associated with shorter OS, while overexpression of TNFAIP8L2 (*P* = 2.69E−2, Fig. 4i) was significantly associated with longer OS in HNC patients. These data indicate that PTX3, TNFAIP6 and TNFAIP8L2 possess important prognostic value.

### The prognostic value of TNFAIP gene alterations in HNC patients

**Figure 5:**
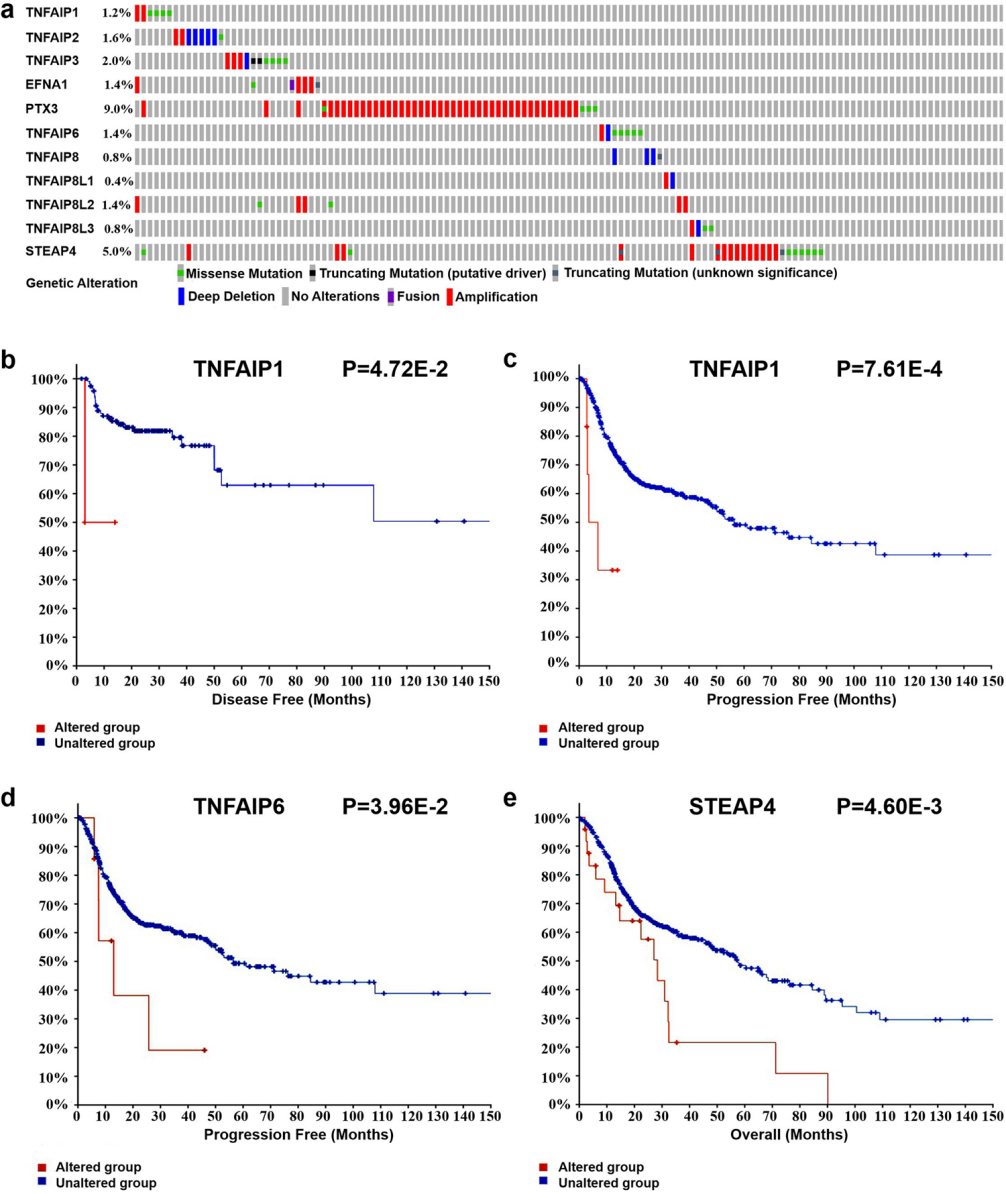
Genetic alterations of TNFAIPs in HNC patients and their effect on prognosis. (**a**) Summary of alterations in TNFAIPs. (**b**) The DFS and (**c**) PFS curves of HNC patients with/without TNFAIP1 alteration. (**d**) The PFS curve of HNC patients with/without TNFAIP6 alteration. (**e**) The OS curve of HNC patients with/without STEAP4 alteration (cBioPortal).

We evaluated genetic alterations of the TNFAIPs in 523 HNSC samples with cBioPortal, and the alteration rates of TNFAIP1, TNFAIP2, TNFAIP3, EFNA1, PTX3, TNFAIP6, TNFAIP8, TNFAIP8L1, TNFAIP8L2, TNFAIP8L3, and STEAP4 were 1.2%, 1.6%, 2.0%, 1.4%, 9.0%, 1.4%, 0.8%, 0.4%, 1.4%, 0.8%, and 5.0%, respectively (Fig. 5a). Moreover, we assessed the impact of genetic alterations on clinical outcome, and the results showed that genetic alterations in TNFAIP1 were significantly associated with shorter disease-free survival (DFS) and progression-free survival (PFS) in HNSC patients (*P* = 4.72E−2 and *P* = 7.61E−4, Fig. 5b, c). Similarly, HNSC patients with genetic alterations in TNFAIP6 and STEAP4 exhibited shorter PFS (*P* = 3.96E−2, Fig. 5d) and worse OS (*P* = 4.06E−3, Fig. 5e) than those without these alterations.

### TNFAIP interaction network analysis, functional annotation and pathway enrichment analysis

**Figure 6:**
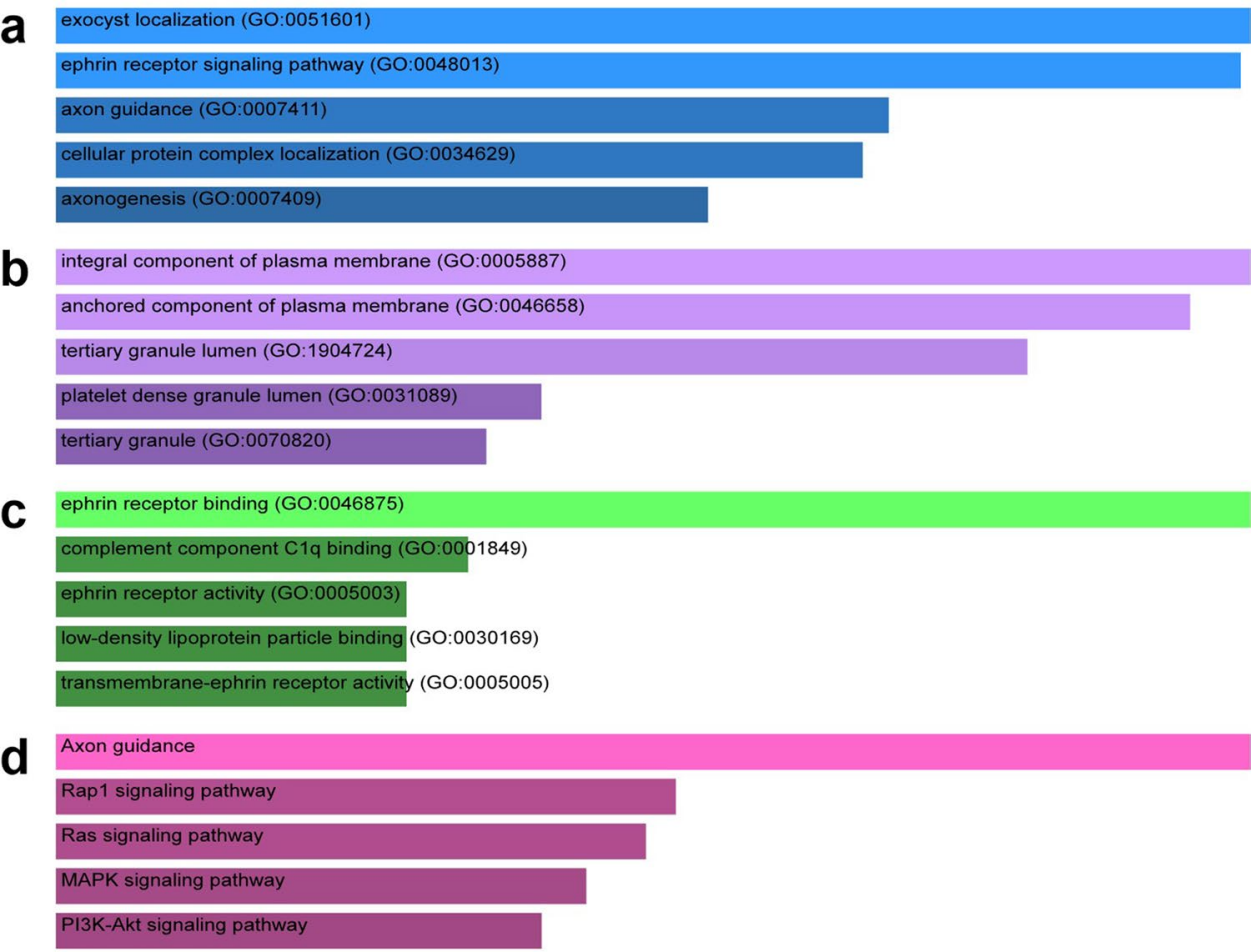
The enrichment analysis of TNFAIPs. (**a**) Bar plot of enriched GO BP terms. (**b**) Bar plot of enriched GO CC terms. (**c**) Bar plot of enriched GO MF terms. (**d**) Bar plot of enriched KEGG pathways^53^ (Enrichr).

Based on the GeneMANIA database, we obtained the interaction network between TNFAIPs and their 20 associated genes, namely, EXOC3L1, EXOC3L4, EXOC3L2, EXOC3, ITIH4, RP11-540D14.8, EFNA5, EFNA4, EFNA2, EFNB3, EFNA3, EFNB2, EFNB1, NPTX1, NPTX2, CRP, OTUD7B, APCS, PTX4, and STEAP1B (Supplementary Figure S1). To further explore the biological function of TNFAIPs, the Enrichr tool was utilized to analyse gene ontology (GO) function and Kyoto Encyclopedia of Genes and Genomes (KEGG) pathway enrichment. GO functional annotation included biological process (BP), cellular component (CC) and molecular function (MF) terms. The top 5 enriched GO terms and KEGG pathways are listed in Fig. 6. GO BP analysis showed that TNFAIPs were significantly enriched in exocyst localization, the ephrin receptor signalling pathway, and axon guidance (Fig. 6a). For CC analysis, the targeted genes were significantly enriched in integral component of plasma membrane, anchored component of plasma membrane, and tertiary granule lumen (Fig. 6b). MF analysis revealed enrichment of ephrin receptor binding, complement component C1q binding, and ephrin receptor activity (Fig. 6c). KEGG pathway enrichment analysis revealed that TNFAIPs were significantly enriched in the Axon guidance, Rap1, Ras, MAPK, and PI3K-Akt signalling pathways (Fig. 6d).

### The correlation between TNFAIPs and infiltrating immune cells in HNC patients

**Figure 7:**
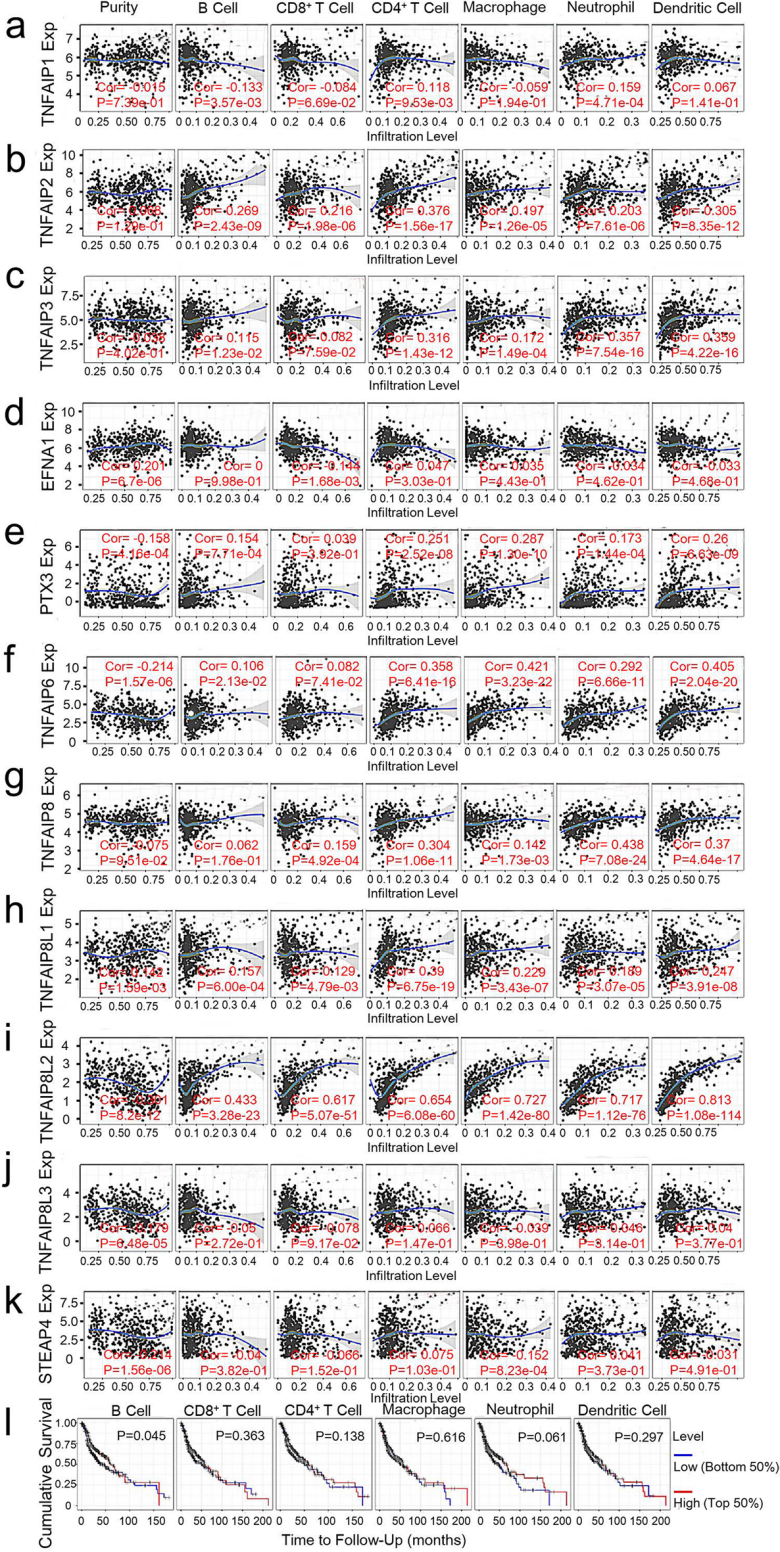
The correlations between immune cell infiltration and TNFAIPs and prognosis. The correlations between the abundance of immune cells and the expression of (**a**) TNFAIP1, (**b**) TNFAIP2, (**c**) TNFAIP3, (**d**) EFNA1, (**e**) PTX3, (**f**) TNAFIP6, (**g**) TNAFIP8, (**h**) TNAFIP8L1, (**i**) TNAFIP8L2, (**j**) TNAFIP8L3, and (**k**) STEAP4 in HNC. (**l**) The OS curve of HNC patients stratified by immune cell infiltration (TIMER).

TNFAIPs may participate in the process of tumour immunomodulation. Accordingly, we performed Tumor IMmune Estimation Resource (TIMER) database analysis to determine the correlation between TNFAIPs and infiltrating immune cells in 522 patients with HNC. TNFAIP1 expression was negatively related to the infiltration of B cells (r = − 0.133, *P* = 3.57E−3) and positively related to the infiltration of CD4^+^ T cells (r = 0.118, *P* = 9.53E−3) and neutrophils (r = − 0.159, *P* = 4.71E−4; Fig. 7a). The expression levels of TNFAIP2, TNFAIP8L1, and TNFAIP8L2 were positively correlated with the infiltration of dendritic cells, neutrophils, macrophages, CD4^+^ T cells, CD8^+^ T cells, and B cells (all *P* < 0.05; Fig. 7b, h, i). However, EFNA1 expression was negatively related to the infiltration of CD8^+^ T cells (r = − 0.144, *P* = 1.68E−3; Fig. 7d). The expression levels of TNFAIP3, PTX3 and TNFAIP6 were positively correlated with the infiltration of dendritic cells, neutrophils, macrophages, CD4^+^ T cells, and B cells (all *P* < 0.05; Fig. 7c, e, f). We also found that high expression of TNFAIP8 was associated with high levels of CD8^+^ T cell (r = 0.159, *P* = 4.92E−4), CD4^+^ T cell (r = 0.304, *P* = 1.06E−11), macrophage (r = 0.142, *P* = 1.73E−3), neutrophil (r = 0.438, *P* = 7.08E−24), and dendritic cell (r = 0.370, *P* = 4.64E−17; Fig. 7g) infiltration. However, there was no significant correlation between TNFAIP8L3 expression and immune cell infiltration (all *P* ≥ 0.05; Fig. 7j). In the end, we found that the infiltration of macrophages was negatively associated with STEAP4 expression (r = − 0.152, *P* = 8.23E−4; Fig. 7k).

We also evaluated the value of infiltrating immune cells in the progression of HNC, and the OS curves of patients stratified according to immune cell abundance are shown in Fig. 7l. High levels of B cell infiltration were significantly associated with longer OS in HNC patients (*P* = 0.045). There was no significant relationship between the infiltration level of other immune cells and prognosis. The above data indicate that PTX3, TNFAIP6, and TNFAIP8L2 are significantly correlated with OS. Therefore, the Cox proportional hazard model was applied, and we corrected for the following confounding factors: B cell infiltration levels and PTX3, TNFAIP6 and TNFAIP8L2 expression levels. The results showed that B cell infiltration levels (*P* = 0.044) and PTX3 expression levels (*P* = 0.003) were significantly associated with the clinical outcome of HNC patients (Supplementary Table S2).

## Discussion

Increasing evidence has shown that TNFAIPs play an important role in the tumorigenesis and progression of HNC^36,37^. Some studies have reported that TNFAIP expression is upregulated in the infiltrating CD4^+^/CD8^+^ T cells of patients with HNC, suggesting that TNFAIPs may be involved in the modulation of tumour progression and immunotherapeutic effects. However, the prognostic value of TNFAIPs in HNC has yet to be fully elucidated.

According to the Oncomine and UALCAN databases, we found that all members of the TNFAIP family were differentially expressed in HNC. Analysis of both databases revealed that the transcript levels of TNFAIP1, TNFAIP3, EFNA1, TNFAIP6 and TNFAIP8 were significantly upregulated, while those of TNFAIP8L3 and STEAP4 were significantly downregulated. In addition, analysis of the Human Protein Atlas indicated that the protein expression levels were consistent with the transcript levels. We also found that the mRNA expression levels of EFNA1, TNFAIP8, and TNFAIP8L3 were closely related to the pathological stage. As HNC progressed, the expression of EFNA1 increased, while that of TNFAIP8 and TNFAIP8L3 decreased. Moreover, our analysis showed that overexpression of PTX3 or TNFAIP6 was associated with poorer OS, while overexpression of TNFAIP8L2 was significantly associated with better OS in HNC patients. These data indicate that differentially expressed TNFAIPs may play a significant role in HNC. Previous studies revealed that distant metastasis-free survival was short in HNC patients with overexpression of TNFAIP2^38^. However, only 95 patients were analysed in that study, and the prognostic value of TNFAIP2 was relatively limited. At present, the prediction of HNC prognosis mainly depends on the TNM stage. However, the TNM stage is based on anatomical information and does not reflect the biological heterogeneity of HNC. In addition, some patients lack TNM stage data in clinical practice; therefore, identification of new biomarkers will compensate for the limitations of TNM stage and help to accurately predict the prognosis of patients.

Malignant tumours are genetic susceptibility diseases, and genetic alterations act as a significant determinant of the development and progression of cancers. With the rapid development of molecular oncology, genetic alteration detection has become increasingly important for research, diagnosis and treatment. In this study, we identified frequent genetic alterations in several TNFAIPs, especially the PTX3 and STEAP4 genes, which had alteration rates of 9% and 5%, respectively. These results may provide novel insights for conducting susceptibility studies and may facilitate early diagnosis and screening for HNC. In addition, our analysis revealed that HNC patients with TNFAIP1 gene alterations had shorter DFS and PFS than those without alterations. Similarly, TNFAIP6 and STEAP4 gene alterations were associated with poorer PFS and OS, respectively, in HNC patients. These data suggest that TNFAIP1, TNFAIP6, and STEAP4 gene alterations possess clinical value in monitoring the prognosis of HNC patients.

GO analysis showed that TNFAIPs were significantly enriched in exocyst localization, ephrin receptor signalling pathway, complement component C1q binding, axon and plasma membrane composition. KEGG pathway analysis revealed that TNFAIPs were significantly enriched in the Axon guidance, Rap1, Ras, MAPK, and PI3K-Akt signalling pathways. The above signalling pathways have vital catalytic roles in the development and progression of malignant diseases; thus, TNFAIPs may promote HNC tumorigenesis through these pathways. KEGG pathway analysis indicated that each member of the TNFAIP family works independently rather than as a complex in the same pathway. However, TNFAIPs may play a synergistic role with other genes in different pathways. It has been reported that PI3K-Akt and NF-κB mediate the transcription response induced by PTX3 by regulating AP-1, resulting in immune escape of HNC^37^. Another study revealed that TNFAIP8L2 participates in regulating the activation of PI3K protein kinase and inhibits the formation of the Ras/RGL complex through competitive binding of the proto-oncogene Ras, thereby inhibiting the downstream Akt signalling pathway of Ras/RGL^39^. The mechanisms of TNFAIPs are complicated and have yet to be fully elucidated. TNFAIP1 could promote proliferation by upregulating caspase-3 and downregulating NF-κB and MMP2 in osteosarcoma cells^18^. TNFAIP2 might promote cancer cell proliferation, invasion and metastasis via the NF-κB, retinoic acid, and Kruppel-like factor 5 signalling pathways^40,41^. A study confirmed that inactivated TNFAIP3 could promote the uncontrolled activation of the NF-κB signalling pathway, which improved cell viability and led to lymphoma^42^. EFNA1-induced EphA1 activation promotes SDF-1 secretion and endothelial progenitor cell chemotaxis to hepatocellular carcinoma through the SDF-1/CXCR4 signalling pathway^43^. Activated NF-κB could activate TNFAIP8 and induce upregulation of the expression of p-Rb, cyclin D1, MMP-1, MMP-2 and VEGFR-2, leading to proliferation, invasion, metastasis and angiogenesis of tumours^44^. TNFAIP8L1 induces tumour cell apoptosis by inhibiting the JNK signalling pathway^44^. TNFAIP8L3 induces cancer cell proliferation and migration and inhibits apoptosis by regulating the NF-κB, PI3K-Akt, and MAPK signalling pathways^44^.

Infiltrating immune cells are an integral part of the tumour microenvironment and play an important role in regulating tumour growth, invasion and metastasis. Previous studies reported that TNFAIP8 is highly expressed in the infiltrating CD4^+^/CD8^+^ T cells of patients with thyroid cancer^36^, suggesting that it might be involved in the occurrence and progression of HNC via the modulation of tumour immune status. Our analysis revealed that the expression levels of TNFAIPs were closely correlated with the infiltration of immune cells (B cells, CD8^+^ T cells, CD4^+^ T cells, macrophages, neutrophils and dendritic cells) in HNC. These data indicate that TNFAIPs are not only prognostic biomarkers but also may reflect the immune status of HNC patients.

Our study still has some limitations; for example, the research data came from a single online database, and another independent cohort is needed to verify the above results in the future. There are some differences among HNC patients due to the different histological types and multiple anatomical sites; therefore, future studies should be refined and revised. In conclusion, this study suggests that several TNFAIPs are differentially expressed in HNC. Moreover, the expression levels of PTX3, TNFAIP6, and TNFAIP8L2 and the genetic alterations of TNFAIP1, TNFAIP6, and STEAP4 greatly impact the clinical outcome of HNC patients, likely by regulating the PI3K-Akt, Ras or other signalling pathways or by regulating tumour immune status. This study may provide novel biomarkers and assist in the design of molecular targeted therapy for HNC.

## Materials and methods

### Ethics statement

Approval by an ethics committee was not required because the current study adhered to online database publication guidelines and data access policies.

### Oncomine

Oncomine (www.oncomine.org) is one of the largest oncogene chip database worldwide, containing 715 gene expression datasets from 86,733 samples^45^. The expression data of TNFAIPs mRNA in HNC were obtained from Oncomine 4.5. A fold change of 2, a gene rank in the top 10%, and *P* < 0.05 were considered as statistically significant in our study. T test was performed to analyse the difference in TNFAIP expression in HNC samples versus normal samples.

### UALCAN

UALCAN (http://ualcan.path.uab.edu) is a comprehensive, user-friendly, and interactive web database for the analysis of oncology data mainly from The Cancer Genome Atlas (TCGA) and MET500^46^. The expression data of TNFAIPs mRNA in HNC were obtained from the “TCGA gene analysis” module and the “HNSC” dataset. T test was performed to analyse the difference in TNFAIP expression, and *P* < 0.05 was considered to indicate statistical significance.

### Human Protein Atlas

The Human Protein Atlas (https://www.proteinatlas.org) provides tissue and cellular distribution information for approximately 26,000 human proteins. The information was obtained via immunoassay techniques (immunohistochemistry, immunofluorescence, and western blotting) to detect proteins expression in 64 cell lines, 48 normal human tissues and 20 tumour tissues^3^. In this study, we compared the expression levels of TNFAIPs between normal and HNSC tissues using immunohistochemical images.

### LinkedOmics

LinkedOmics (http://www.linkedomics.org/) is a publicly available portal that provides comprehensive multi-omics data analysis of 32 TCGA cancer types^47^. In this study, “TCGA-HNSC” was selected as the cancer type module, “RNA-seq” and “clinical” were selected as the data type module. The relationships between gene expression levels and clinicopathological parameters and OS were analysed by nonparametric test and Cox regression analysis, respectively. *P* < 0.05 was considered to indicate statistical significance.

### cBioPortal

cBioPortal (www.cbioportal.org) is a comprehensive web resource developed by Memorial Sloan-Kettering Cancer Center that contains information from 287 cancer studies. This resource can be applied to visualize and analyse multidimensional cancer genomics datasets^48^. This study analysed genetic alterations of TNFAIPs in 523 patients with HNSC (TCGA, Pan-Cancer Atlas). In addition, the Kaplan–Meier method and log-rank test were used to analyse the relationship between genetic alterations and OS/DFS. *P* < 0.05 was considered to indicate statistical significance.

### GeneMANIA

GeneMANIA (http://www.genemania.org) is an open database used to analyse the correlations among genes^49^. It can find other genes that are related to a set of input genes using a very large set of functional association data, which include protein and genetic interaction, pathway, co-expression, co-localization and protein domain similarity data. In this study, TNFAIPs were inputted to obtain the related genes and their association data.

### Enrichr

Enrichr (http://amp.pharm.mssm.edu/Enrichr/) is a commonly used GO functional annotation and KEGG pathway enrichment analysis tool^50^. GO functional annotation includes BP, CC and MF terms. In this study, TNFAIPs and their associated genes were inputted in the “analyse” module. *P* < 0.05 was considered to indicate statistical significance.

### TIMER

TIMER (https://cistrome.shinyapps.io/timer/) is a web server for the analysis of infiltrating immune cells and their clinical impact^51^. In this study, the “gene” module was used to evaluate the relationship between TNFAIP expression and infiltrating immune cells, and the “survival” module was used to analyse the relationship between OS and infiltrating immune cells/TNFAIPs. The Kaplan–Meier method and the log-rank test were utilized in this study, and *P* < 0.05 was considered to indicate statistical significance.

## Supplementary Information

Supplementary Information.

## Data Availability

The data that support the findings of this study are available in The Cancer Genome Atlas (TCGA) datasets at https://www.cancer.gov/about-nci/organization/ccg/research/structural-genomics/tcga.
