# Supplementary material for: Comprehensive analysis of the expression and prognosis for TNFAIPs in head and neck cancer
Source: Sci Rep. 2021 Aug 3;11:15696. doi: 10.1038/s41598-021-95160-x (PMC8333337; doi:10.1038/s41598-021-95160-x)
Supplement: Supplementary file 1 — Supplementary Information. [file 41598_2021_95160_MOESM1_ESM.pdf]

Comprehensive analysis of the expression and prognosis for TNFAIPs in head and neck cancer

Gaochen Lan[1, 3] , Xiaoling Yu[1, 3], Xin Sun[2], Wan Li[1], Yanna Zhao[1], Jinjian Lan[1], Xiaolong Wu[1], Ruilan Gao[1*]

[1]Institute of Hematology Research, the First Affiliated Hospital of Zhejiang Chinese Medical University, Hangzhou, 310006, China.

[2]Department of Oncology, Zhejiang Provincial People's Hospital, Hangzhou, 310014, China.

[3]These authors contributed equally: Gaochen Lan and Xiaoling Yu.

[*]Corresponding author: Dr. Ruilan Gao, the First Affiliated Hospital of Zhejiang Chinese Medical University, 54 Youdian Road, Hangzhou, 310006, China. E-mail: 19843009@zcmu.edu.cn.

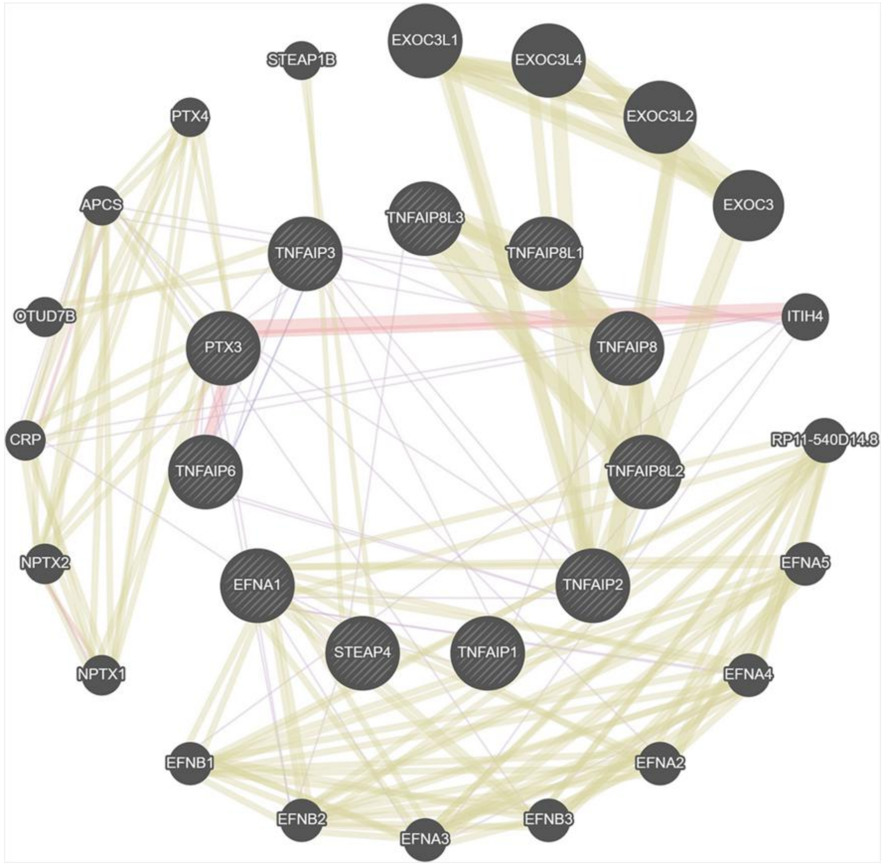

Supplementary Figure S1. The interaction network between TNFAIPs and their 20 associated genes. The red connection indicates physical interactions, the purple connection indicates co-expression, the blue connection indicates co-localization, and the yellow connection indicates protein domain similarity (GeneMANIA).

Variables	NO.	TNFAIP1		TNFAIP2		TNFAIP3		EFNA1		PTX3		TNFAIP6		TNFAIP8		TNFAIP8L1		TNFAIP8L2		TNFAIP8L3		STEAP4	
		mRNA	*P* value	mRNA	*P* value	mRNA	*P* value	mRNA	*P* value	mRNA	*P* value	mRNA	*P* value	mRNA	*P* value	mRNA	*P* value	mRNA	*P* value	mRNA	*P* value	mRNA	*P* value
Pathology_T_stage			0.340		6.67E-01		2.81E-01		4.09E-04		3.81E-01		4.61E-01		3.42E-02		1.30E-01		1.68E-03		9.16E-03		7.46E-02
T1-T2	179																						
T3-T4	269							↑						↓				↓		↓			
Pathology_N_stage			7.03E-04		2.54E-01		9.15E-02		9.75E-02		2.15E-01		5.75E-01		1.77E-01		8.07E-01		4.39E-02		3.12E-02		3.93E-03
N0	173																						
N1-3	238	↓																↑		↓		↓	
Pathology_M_stage			9.39E-01		2.78E-01		0.148		5.55E-01		2.02E-01		1.38E-01		9.85E-01		1.77E-01		0.238		9.85E-01		8.34E-01
M0	181																						
M1	1																						
Pathologic stage			3.00E-01		4.65E-01		6.97E-01		2.68E-02		7.44E-01		5.96E-01		2.77E-02		2.53E-01		0.299		6.48E-03		1.83E-01
I-II	100																						
III-IV	339							↑						↓						↓			
Radiotherapy			2.65E-02		2.02E-02		7.01E-02		3.60E-03		2.51E-01		0.272		8.72E-01		3.87E-01		9.57E-01		2.57E-02		6.05E-03
No	155																						
Yes	296	↓		↑				↑												↓		↓	

Supplementary Table S1. The relationship between the mRNA levels of TNFAIPs and clinicopathological parameters in HNC patients; ↑ and ↓ represents upregulation and downregulation, respectively (LinkedOmics).

	Coef	HR	95%CI_l	95%CI_u	*P*-value	Sig
B cell	-2.124	0.120	0.015	0.947	0.044	*
PTX3	0.178	1.195	1.061	1.346	0.003	**
TNFAIP6	0.039	1.040	0.919	1.177	0.536	
TNFAIP8L2	-0.095	0.909	0.767	1.078	0.273	

Supplementary Table S2. The cox proportional hazard model of immune cell infiltration levels and TNFAIP expression levels in HNC.

HR, hazard ratio; CI_l, lower confidence interval; CI_u, upper confidence interval; *P<0.05, **P<0.01 (TIMER).
